# Vitronectin and dermcidin serum levels predict the metastatic progression of AJCC I–II early‐stage melanoma

**DOI:** 10.1002/ijc.30202

**Published:** 2016-06-11

**Authors:** Idoia Ortega‐Martínez, Jesús Gardeazabal, Asier Erramuzpe, Ana Sanchez‐Diez, Jesús Cortés, María D. García‐Vázquez, Gorka Pérez‐Yarza, Rosa Izu, Jose Luís Díaz‐Ramón, Ildefonso M. de la Fuente, Aintzane Asumendi, María D. Boyano

**Affiliations:** ^1^ Department of Cell Biology and Histology, Faculty of Medicine and DentistryUniversity of the Basque Country (UPV/EHU)LeioaBizkaiaSpain; ^2^ Department of Dermatology, Ophthalmology and Otorhinolaryngology, Faculty of Medicine and DentistryUniversity of the Basque Country (UPV/EHU)LeioaBizkaiaSpain; ^3^ BioCruces Health Research InstitutePlaza De Cruces S/N, BarakaldoBizkaiaSpain; ^4^ Ikerbasque: The Basque Foundation for ScienceBilbaoSpain; ^5^ Institute of Parasitology and Biomedicine Lopez‐NeyraParque Tecnológico Ciencias De La Salud, Avenida Del Conocimiento S/N, ArmillaGranadaSpain

**Keywords:** melanoma, serum markers, prognosis, serum amyloid A, clusterin, plakoglobin, dermcidin, vitronectin

## Abstract

Like many cancers, an early diagnosis of melanoma is fundamental to ensure a good prognosis, although an important proportion of stage I–II patients may still develop metastasis during follow‐up. The aim of this work was to discover serum biomarkers in patients diagnosed with primary melanoma that identify those at a high risk of developing metastasis during the follow‐up period. Proteomic and mass spectrophotometry analysis was performed on serum obtained from patients who developed metastasis during the first years after surgery for primary tumors and compared with that from patients who remained disease‐free for more than 10 years after surgery. Five proteins were selected for validation as prognostic factors in 348 melanoma patients and 100 controls by ELISA: serum amyloid A and clusterin; immune system proteins; the cell adhesion molecules plakoglobin and vitronectin and the antimicrobial protein dermcidin. Compared to healthy controls, melanoma patients have high serum levels of these proteins at the moment of melanoma diagnosis, although the specific values were not related to the histopathological stage of the tumors. However, an analysis based on classification together with multivariate statistics showed that tumor stage, vitronectin and dermcidin levels were associated with the metastatic progression of patients with early‐stage melanoma. Although melanoma patients have increased serum dermcidin levels, the REPTree classifier showed that levels of dermcidin <2.98 μg/ml predict metastasis in AJCC stage II patients. These data suggest that vitronectin and dermcidin are potent biomarkers of prognosis, which may help to improve the personalized medical care of melanoma patients and their survival.

## Abstract 2

**What's new?**

The discovery of serum biomarkers capable of predicting metastatic risk during post‐operative follow‐up in early‐stage primary melanoma patients could significantly benefit patient prognosis and survival. The present study identifies two promising biomarker candidates: vitronectin and dermcidin. Analysis and comparison of serum proteins in melanoma patients who either remained disease‐free or developed metastases in the years after surgery to remove primary tumors revealed a strong association between metastatic progression of early‐stage melanomas (AJCC stage III) and vitronectin and dermcidin serum levels. Further study of these proteins could open up new opportunities in the effort to improve long‐term survival among melanoma patients.

The prognosis and survival of patients with melanoma depends mainly on the early diagnosis of the disease. Patients with tumors <1 mm thick and with no lymph node involvement have a high likelihood of being cured after complete surgical excision of the tumor. However, there is an important proportion of such patients who develop metastasis during the first years of follow‐up.^1^

The present staging system for melanoma takes into account Breslow thickness, ulceration, mitotic rate and the presence of regional or distant metastases to stratify patients into heterogeneous groups, although they have a widely variable outcome.^2^ Classically, melanoma is divided into four stages according to the American Joint Committee on Cancer (AJCC) classification^3^: stage I–II involves primary tumors of distinct thickness; stage III is defined by locoregional spread of the disease and stage IV where melanoma cells spread to distant organs (metastasize). Surgical resection of melanoma is the curative treatment in the early stages (stage I–II), yet some high‐risk patients at this stage subsequently develop a fatal metastasis.^4^

Protein biomarkers that are associated with the malignant properties of melanoma cells could help better classify melanoma and stratify patients in function of their risk of metastasis in order to better select surgical and adjuvant treatments. There are currently very few serological biomarkers available to help clinically diagnose early‐stage melanoma and to tailor therapy or predict metastasis. The only biomarker included in the AJCC staging system is lactate dehydrogenase (LDH), the strongest independent prognostic factor for stage IV melanoma,^5^ although an important increase in serum S100β levels has been associated to metastatic progression, irrespective of stage.^6^

We previously demonstrated that serum levels of the soluble IL‐2 receptor (sIL‐2R), sICAM‐1 and Interleukin‐10 (IL‐10) are associated with poor prognosis in patients with melanoma.^7^, ^8^ Other serological markers have also been shown to be associated with the advanced stages of the disease, including MIA, tyrosinase, VEGF, IL‐6 and IL‐8.^9^ However, there are not yet consolidated serological biomarkers in clinical use to predict metastasis in AJCC stage I–II melanoma patients. Thus, the aim of this work was to identify serological biomarkers that can be used at the time of primary melanoma diagnosis to identify early‐stage patients at high risk of developing metastasis during follow‐up.

## Material and Methods

### Patients and controls

The study focused on 348 patients, all of them with histologically confirmed malignant melanoma: 210 women and 138 men; mean age 52.24 years (range 17–88: see Table 1 for full patient characteristics). The patients were untreated, other than primary surgery, and they were free of infections as judged by clinical evaluation and the absence of infectious blood parameters. Serum was obtained from venous blood samples provided by all the subjects, aliquots of which were stored at −80°C. After surgery of the primary tumor, the patients were given a medical examination every 3 months during the first 2 years and every 6 months thereafter for a 5‐year total follow‐up. After the fifth year, they received an annual revision up to the tenth year postsurgery. Those patients who developed metastasis during the follow‐up period were then examined every 3 months for the following 2 years. The presence or absence of metastasis was assessed in all patients by physical examination, as well as laboratory and radiological testing. The appearance of metastasis was always confirmed by radiographic examination and/or computed tomography scanning. Disease stages were classified according to the AJCC. The melanoma patients at early disease stages (I and II) were divided into two groups according to disease prognosis: patients with good prognosis (10‐year disease‐free survival after surgery of the primary tumor) and metastatic patients (patients who developed metastasis during the first 2 years of follow‐up after surgery). Of the 271 stage I–II patients, 67 (24.7%) developed metastasis during follow‐up. A group of “*in situ*” melanoma patients and a group of advanced stage of melanoma patients (III and IV, respectively) were also included in the study. All serum samples tested were collected at the moment of melanoma diagnosis before surgery.

**Table 1:** Patient characteristics and controls

	Number	Gender (male/female)		
Controls	100	32/68		
Patients	348	138/210		
**Tumor location**				
Head/neck	45	24/21		
Trunk	122	75/47		
Upper limb	38	10/28		
Lower limb	104	18/86		
Hand/foot	35	14/21		
Unknown	4	–		
**Stage** a				
0 *(in situ*)	45	13/32		
I	184	63/121		
II	87	42/45		
III	27	15/12		
IV	5	5/0		
**Metastasis**	***N* (%)**	**In transit**	**Nodal**	**Distant**
*In situ*	0 (0)			
I/II stages	67 (24.7)	6	17	44
III/IV stages	32 (100)	0	10	22

a The American Joint Committee of Cancer (AJCC) staging system for melanoma was used.

The control group consisted of 100 healthy donors: 68 women and 32 men with a mean age of 32.04 years (range 21–57).

### Ethics statement

This study was approved by the Ethics Committees at the Cruces and Basurto University Hospitals (Bizkaia, Spain). Written informed consents were obtained from all the subjects and the collection of melanoma samples is recorded at the Spanish Health Institute Carlos III (number: C.0002121).

### Proteomic analysis

Serological proteins related to the metastatic progression of melanoma in early clinical‐stage patients were identified by analyzing ten serum samples from AJCC stage IIA melanoma patients: five melanoma patients who developed metastasis during the first 2 years of follow‐up (three women and two men, mean age 62.8 years, range 46–84) and five patients who remained free of tumor disease for 10 years of clinical follow‐up (three men and two women, mean age 57.6, range 23–78).

### Protein enrichment in serum samples

To enhance the detection of proteins before the proteomic analysis and in particular, to alleviate the suppression of the signal from less abundant proteins by those more abundant proteins, such as albumin and immunoglobulins, each serum sample was prefractioned using the ProteoMinerTM kit (Bio‐Rad Laboratories, Hercules, CA) according to the manufacturer's protocol. Prefractioned serum samples were stored at −20°C until use. The protein concentration of each sample was determined by the Bicinchoninic Acid method^10^ using bovine serum albumin (BSA) to generate a standard curve (bicinchoninic acid solution and BSA were obtained from Sigma‐Aldrich Quimica S.A., Madrid, Spain).

### 2D gel electrophoresis

The first dimension of the 2D gel electrophoresis (2‐DE) separation of proteins involved their isoelectric focusing (IEF). Immobilized pH gradient (IPG) strips (11 cm, pI 3–10) were actively rehydrated at 50 V for 12 h and the sample (100 μg) was loaded on each gel after diluting up to 200 μl with rehydration buffer (Bio‐Rad) and 1.2% Destreak Reagent (GE Healthcare, Uppsala, Sweden). Dehydration and oxidation of the strips were prevented by covering them with mineral oil (Bio‐Rad) and the electrofocusing was performed in the PROTEIN® IEF Cell (Bio‐Rad) at 20°C under the following regime: 250 V (20 min), 8,000 V (2,5 h), 8,000 V (25,000 V/h). Before molecular weight resolution of the proteins by sodium dodecyl sulphate‐polyacrylamide gel electrophoresis (SDS‐PAGE), the IPG strips were incubated on a rocking table for 10 min at room temperature in SDS equlibration buffer [6 M urea, 20% glycerol, 2% SDS, 0.375 M Tris‐HCl (pH 8.8) and 2% DTT] to reduce the disulfide bonds in the proteins. The strips were then incubated for 20 min in the same solution containing 2.5% iodoacetamide instead of DTT to alkylate the free cysteine residues in the focused proteins. The proteins were resolved on 12% BisTris Criterion precast SDS‐PAGE gels (Bio‐Rad), running the ten gels simultaneously at 200 V and 25°C in Crierium Dodeca Cell (Bio‐Rad) until the bromophenol blue dye front had reached the bottom of the gel.

### Protein visualization and image analysis

For image analysis, gels were stained with fluorescent SYPRO Ruby Protein Gel Stain (Bio‐Rad) according to the manufacturer's instructions. Fluorescent stained gels were digitalized in a Molecular Imager FX scanner, obtaining images with Quantity One 4.2.3 software (Bio‐Rad) and analyzing them with ProgenesisSameSpots software (Nonlinear Dynamics, Newcastle, UK). The gel patterns from each independent analysis were matched and the relative volume of each spot (%V) in the two gel sets were compared (disease‐free and metastasis progression). Spots were considered to be expressed distinctly if the difference in the relative volume of each spot was significant at *p* < 0.05. Gels were stored in deionized water at 4°C and to visualize the spots of interest for further analysis, silver staining was performed as described by Rabilloud,^11^ with minor modifications.

### In‐gel digestion

Selected protein spots were excised manually from the gel and subjected to in‐gel tryptic digestion according to Shevchenko *et al*.,^12^ with minor modifications. Gel spots were destained by washing in 50 mM K_3_[Fe(CN)_6_] and 15 mM Na_2_O_3_S_2_ and then in MilliQ water. Destained gel pieces were swollen in digestion buffer containing 50 mM NH_4_HCO_3_ and 12.5 ng/μl proteomic grade trypsin (Roche, Basel, Switzerland) and digested overnight at 37°C. The supernatant was recovered and the peptides were extracted twice from the gel: first with 25 mM NH_4_HCO_3_ and acetonitrile (Romil, Cambridge, UK) and then with 0.1% trifluoroactic acid (Merck, Darmstadt, Germany) and acetonitrile (Romil). The supernatants recovered and the peptides extracted were pooled, dried in a SpeedVac (Thermo Fisher Scientific, Waltham, MA), redissolved in 10 μl of 0.1% formic acid (Merck), sonicated for 5 min and analyzed directly by LC‐MS/MS or stored at −20°C.

### LC‐MS/MS analysis

LC‐MS/MS spectra were acquired on a SYNAPT HDMS mass spectrometer (Waters, Milford, MA) with a nanoAcquity UPLC System interface (Waters). Each sample (8 μl) was loaded onto a Symmetry 300 C18 (180 μm × 20 mm) precolumn (Waters) and washed with 0.1% formic acid for 3 min at a flow rate of 5 μl/min. The precolumn was connected to a BEH130 C18 column (75 μm × 200 mm, 1.7 μm: Waters) equilibrated in 3% acetonitrile and 0.1% formic acid. Peptides were eluted directly onto a NanoEase Emitter (Waters) with a 30 min linear gradient of 3–60% acetonitrile. The capillary voltage was set to 3,000–3,500 V and the data‐dependent MS/MS acquisitions were performed on precursors with charge states of 2, 3 or 4 over a survey *m*/*z* range of 350–1,990.

### Protein identification

The spectra were processed using VEMS^13^ and searched against SwissProt database (versions 56.7 or 57.2) using Mascot (Matrixscience) to identify the proteins using the following parameters: carbamidomethylation of cysteines as fixed modification, oxidation of methionines as variable modification, 25–50 ppm peptide mass tolerance, 0.05–0.1 Da fragment mass tolerance and one missed cleavage. Database entries were restricted to *Homo sapiens*.

### Detection of serum amyloid A (SAA), clusterin (CLU), plakoglobin (PG), vitronectin (VN) and dermcidin (DCD) levels in the serum of melanoma patients and healthy controls

Serum SAA, CLU, PG, VN and DCD concentrations were measured using a sandwich enzyme immunoassay. Commercially available ELISA kits were used according to the manufacturers' instructions (SAA kit from Invitrogen, Carlsbad, CA; CLU kit from ALPCO Diagnostics, Salem, NH; JUP kit from Uscn Life Science, Wuhan, China and VN and DCD kits from Cusabio Biotech, Wuhan, China). Serum SAA, CLU, VN and DCD concentrations were expressed in µg/ml and PG concentrations in ng/ml. The sensitivity of the kits were 4 ng/ml for SAA, 1 ng/ml for CLU, 0.134 ng/ml for PG, 0.39 ng/ml for VN and 0.16 ng/ml for DCD. Lower concentrations were regarded as undetectable and the normal levels of all the proteins were defined by their measurement in healthy controls.

### Statistical analysis and classification

The level of statistical significance between the sample means was determined through parametric (Student's *t*‐test) and nonparametric tests (Mann–Whitney *U*‐test). Statistical analyses were performed using the SPSS statistical software and a *p* value below 0.05 was considered statistically significant. The cumulative survival rates were calculated using the Kaplan–Meier methods and the statistical significance of differences was determined by using the log‐rank test (*p* < 0.05). Logistic proportional hazards regression models were used to define predictive factors linked to metastatic progression, evaluating age, sex, stage, metastatic evolution, disease‐free interval and SAA, CLU, PG, VN and DCD serum levels as independent factors. We defined the disease‐free interval as the period (months) between surgery for the primary tumor and the appearance of metastasis. Patients with a follow‐up <24 months were excluded from the analysis. Disease‐free patients at the end of the follow‐up period were considered censored observations. A relative risk (RR) analysis was also performed to quantify the association of serum markers with metastatic progression.

Different classification methods were applied using the Waikato Environment for Knowledge Analysis (WEKA),^14^ applying different classification algorithms and using different parameters to define the classification rules to separate a metastatic response from a nonmetastatic response. In particular, we made use of the following classifiers: a classifier based on minimal cost‐complexity pruning (implemented in WEKA as SimpleCART); a naive Bayes classifier (implemented as NBtree); a classifier based on a reduced error pruning tree (implemented as REPTree); a classifier using a C4‐5 decision tree (implemented as J48); a classifier using the minimum‐error attribute for prediction (implemented as OneR) and a logistic regression and a linear regression classifier. The best classification performance was achieved by REPTree using a tenfold cross‐validation test. The REPTree classifier is a fast‐decision algorithm that builds a decision tree using adaptive information gain by reduced‐error pruning.

## Results

### Patient characteristics

A total of 348 melanoma patients were studied here, of whom 210 were women and 138 men, with a mean age of 52.24 years. Melanoma occurred more frequently in women than in men and the majority of primary tumors developed on the trunk (*n* = 122) or lower limb (*n* = 104). Of the 348 melanoma patients studied, 45 (12.9%) were classified as melanoma *in situ* at the time of diagnosis, 271 (77.8%) at stages I and II, 27 (7.7%) at stage III and only five (1.4%) patients were considered to be stage IV. Despite the fact that most of the melanoma patients were in an early disease state at the time of diagnosis (AJCC stages I and II), 67 (24.7%) developed metastasis during follow‐up, *i.e*., about one in four patients diagnosed with early‐stage melanoma developed metastasis during the follow‐up and most of them developed distant metastasis (Table 1).

### Proteomic profile

The proteomic profiles of the serum from ten melanoma patients diagnosed at an early stages of the disease (stage IIA) were analyzed by 2‐DE. These melanoma patients were divided into two groups according to the clinical outcome of the disease and their serum proteomic profiles were compared: patients 10 years disease‐free after surgery of the primary tumor and patients who developed metastasis during the first 2‐year follow‐up. When analyzed with Progenesis SameSpots software, 19 protein spots were identified that differed significantly in terms of their intensity of expression (*p* < 0.05). The protein spots with at least a 60% difference in expression were selected for further LC‐MS/MS analysis, allowing a total of 42 proteins to be identified using the MASCOT software (Table 2).

**Table 2:** Summary of the proteins identified

SwissProt entry	Protein	Score	No. of peptides	MW (kDa)	PI
P00734	Prothrombin	67	3	71.47	5.64
P01009	Alpha‐1‐antitrypsin	655	24	46.87	5.37
P35542	Serum amyloid A4	82	9	14.85	9.27
P0C0L4	Complement C4A	741	41	194.24	6.65
P0C0L5	Complement C4B	47	2	194.17	6.89
P01024	Complement C3	1,185	33	188.56	6.02
P04004	Vitronectin	119	5	55.06	5.55
P02647	Apolipoprotein A1	331	16	30.75	5.56
P02652	Apolipoprotein A2	128	4	11.28	6.26
P09871	Complement c1s subcomponent	35	1	78.17	4.86
P04003	C4b‐binding protein alpha chain	42	2	69.04	7.15
P10909	Clusterin	231	7	53.03	5.89
P01871	Ig mu chain C region	96	3	49.96	6.35
P01857	Ig gamma‐1 chain C region	231	8	36.59	8.46
P01859	Ig gamma‐2 chain C region	46	1	36.50	7.66
P01842	Ig lambda chain C region	139	4	11.40	6.92
P01714	Ig lambda chain V‐III region SH	34	2	11.50	6.02
P01834	Ig kappa chain C region	64	1	11.77	5.58
Q9UMS4	Pre‐mRNA processing factor 19	30	1	55.60	6.14
Q15485	Ficolin 2	146	4	34.43	6.31
P01019	Angiotensinogen	31	4	53.40	5.87
P61626	Lysozyme C	34	1	16.98	9.38
Q86SJ6	Desmoglein 4	64	1	11.46	4.42
P14923	Junction plakoglobin	97	2	82.43	5.75
P15924	Desmoplakin	60	2	334.02	6.44
O94833	Bullous pemphigoid antigen 1, isoforms 6/9/10	30	1	593.76	5.49
P81605	Dermcidin	82	2	11.39	6.08
P50213	Isocitrate dehydrogenase [NAD] subunit alpha, mitocondrial	36	1	40.02	6.47
P00338	Lactate dh A chain	40	1	36.95	8.44
Q01968	Inositol polyphosphate 5 phosphatase OCRL‐1	29	1	105.39	6.13
P02768	Serum albumin	227	12	71.31	5.92
O43933	Peroxisome biogenesis factor 1	41	2	143.80	5.91
P02787	Serotransferrin	102	4	79.28	6.81
P62805	Histone H4	55	3	11.36	11.36
P33778	Histone H2B type 1B	27	2	13.94	10.31
P02746	Complement c1q subcomponent subunit B	73	1	26.67	8.83
Q8TF66	Leucine‐rich repeat‐containing protein 15	32	1	65.23	6.24
P01008	Antithrombin‐III	51	3	53.02	6.32
Q99460	26s Proteasome non‐ATPase regulatory subunit 1	27	1	10.67	5.25
P04196	Histidine‐rich glycoprotein	85	2	60.51	7.09
Q96M02	Uncharacterized protein C10 or f90	29	1	79.00	9.21
P04279	Semenogelin‐1	104	2	52.15	9.30

Score: The ion score is −10*log(*p*), where *p* is the probability that the observed match is a random event. Individual ion scores > 26 indicate identity or extensive homology (*p* < 0.05). Peptides: the number of peptides identified matching the protein. MW (kDa): molecular weight of the protein expressed in kDa.

**Figure 1:**
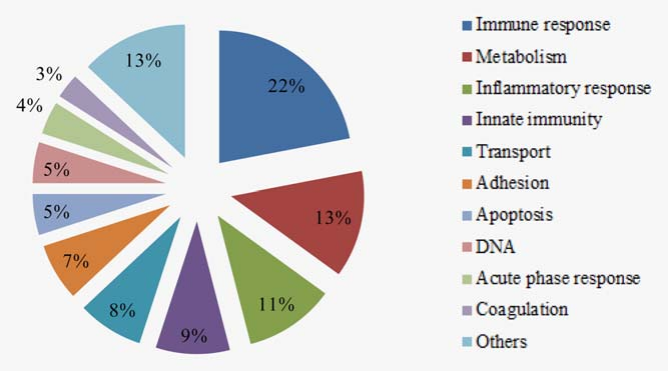
Classification of proteins based on their biological function. The database used as a reference for the functional classification of the proteins identified was UniProtKB (www.expasy.org).

The proteins differentially expressed in melanoma patients were involved in different biological activities and processes, and they were classified into different groups according to their activity and the biological processes in which they are involved (Fig. 1). The activity of most of the proteins seen to be differentially expressed in melanoma patients was related to immune responses (22%), metabolism (13%) and inflammatory responses (11%) and to a lesser extent innate immunity (9%), transport (8%), adhesion (7%), apoptosis (5%), DNA (5%), acute‐phase response (4%) and coagulation (3%).

Among the proteins identified, five were selected for validation by ELISA as potential serum biomarkers of prognosis and disease outcome in the 348 melanoma patients and 100 healthy controls: two apolipoproteins related to inflammatory and immune responses, SAA and CLU; two proteins involved in adhesion, PG and VN and an antimicrobial protein, DCD.

### Serum levels of SAA, CLU, PG, VN and DCD

The accumulation of the five proteins in serum was independent of the age of the healthy controls and melanoma patients (*p* = 0.469), and it did not vary between the sexes (*p* = 0.272). There were significant differences between melanoma patients and the healthy controls in the serum levels of the two apolipoproteins related to immune and inflammatory responses (Table 3), with higher levels of SAA (26.8 ± 1.2 µg/ml) and CLU (24.3 ± 0.6 µg/ml) in the serum of melanoma patients than in that of healthy individuals (12.6 ± 1.4 µg/ml and 15.6 ± 0.5 µg/ml, respectively). There were significantly higher levels of the adhesion proteins assayed in melanoma patient's serum than in that from the controls: 1.3 ± 0.1 ng/ml and 0.82 ± 0.1 ng/ml of PG in serum, respectively, and 14.1 ± 0.4 µg/ml and 9.7 ± 0.7 µg/ml of VN, respectively. Antimicrobial DCD protein serum levels were also significantly higher in melanoma patients (2.8 ± 0.06 µg/ml) than in healthy individuals (2.1 ± 0.1 µg/ml). With regard to tumor stage, in the group of patients at stage 0 (*in situ*) the serum levels of the five proteins were similar to those of the controls, except for the significantly higher levels of serum SAA (*p* < 0.001). Moreover, and although they differed from the controls, no significant differences were found among any of the tumor histopathological stages for any of the serum values (Table 3).

**Table 3:** Serum SAA, CLU, PG, VN and DCD levels in patients with melanoma and in the healthy controls

	*N*	SAA (μg/ml)	CLU (μg/ml)	PG (ng/ml)	VN (μg/ml)	DCD (μg/ml)
Controls	100	12.6 ± 1.4	15.6 ± 0.5	0.82 ± 0.1	9.7 ± 0.7	2.1 ± 0.1
Patients	348	26.8 ± 1.2^b^	24.3 ± 0.6^b^	1.28 ± 0.1^b^	14.1 ± 0.4^b^	2.8 ± 0.06^b^
Stage^a^						
0	45	25.9± 2.8^b^	13.2 ± 1.0	0.4 ± 0.2^b^	10.3 ± 0.9	2.3 ± 0.1
IA	112	25.9± 2.1	27.2 ± 1.1	1.4 ± 0.1	13.7 ± 0.7	2.6 ± 0.1
IB	72	22.5± 2.5	22.7 ± 1.2	1.6 ± 0.2	15.7 ± 1.0	2.7 ± 0.1
IIA	53	29.0± 3.2	24.8 ± 1.8	1.3 ± 0.2	16.0 ± 1.0	3.0 ± 0.1
IIB	25	30.0± 3.7	31.8 ± 2.4	1.6 ± 0.5	16.1 ± 1.3	3.1 ± 0.2
IIC	9	35.9± 6.3	21.3 ± 1.9	1.4 ± 0.4	15.7 ± 2.7	2.6 ± 0.3
IIIA	13	29.9± 5.9	31.3 ± 3.5	1.3 ± 0.2	14.1 ± 2.0	4.3 ± 0.3
IIIB	7	47.0 ± 11.9	26.7 ± 5.9	1.2 ± 0.2	8.5 ± 1.1	2.8 ± 0.3
IIIC	7	27.3± 6.6	24.0 ± 4.1	1.7 ± 0.5	10.8 ± 2.8	2.5 ± 0.3
IV	5	22.8 ± 10.2	34.4 ± 5.1	2.5 ± 0.9	13.4 ± 2.9	4.1 ± 0.5

a The AJCC staging system for melanoma was used. The data represent the mean ± SE.

b *p* < 0.01, significant differences *versus* controls.

**Figure 2:**
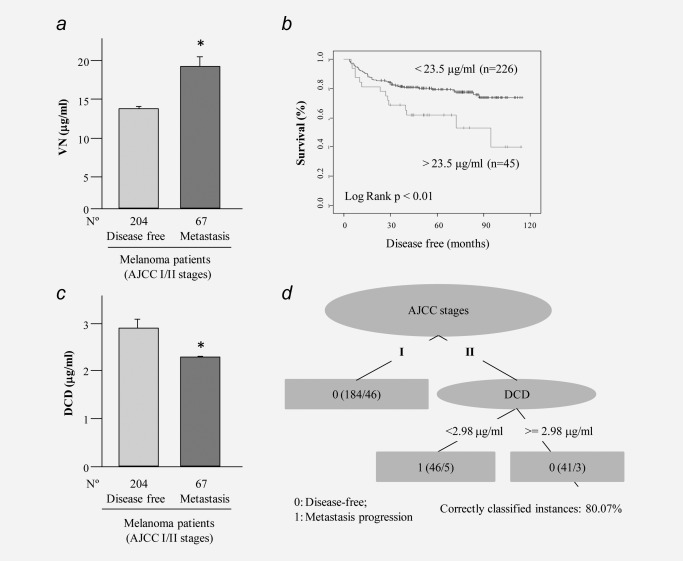
VN and DCD serum levels in early‐stage melanoma patients. (*a*) VN serum levels in stage I–II patients. Patients were divided according to tumor progression as disease‐free patients (more than 10 years disease‐free) and metastatic (patients who developed metastasis during the follow‐up). (*b*) Disease‐free survival curves for melanoma patients relative to their serum VN levels. Stage I–II patients were divided into two groups according to the mean + 2 SD of the VN level in the control group (23.5 µg/ml). (*c*) DCD serum levels in stage I–II patients. Patients were divided as described above. (*d*) Decision‐tree algorithm classified stage II patients relative to their DCD serum levels. A low DCD concentration (<2.98 μg/ml) has an 80% accuracy in predicting metastasis for stage II melanoma patients.

The amounts of the distinct proteins in serum differed in function of the clinical disease outcome, particularly in the case of VN and DCD. VN serum levels were significantly higher in early‐stage melanoma patients who developed metastasis during the first 2 years of the follow‐up (Fig. 2 *a*). Moreover, the survival analysis showed that VN serum levels were related to a poor prognosis of patients diagnosed at early stages. The division of stage I–II patients in two groups in function of their serum VN levels manifested that patients with VN levels below 23.5 µg/ml (mean + 2 SD of the VN level in the control group) presented greater survival (log rank < 0.01) than those with greater than 23.5 µg/ml VN in the serum (Fig. 2 *b*). Moreover, a RR analysis was performed to quantify how strongly VN serum levels were associated with the development of metastasis by patients with early‐stage melanoma. Patients with VN serum levels higher than 23.5 µg/ml at the moment of diagnosis were 2.8 times more likely to develop metastases during the follow‐up (RR = 2.810: 1.305 ≤ RR ≤ 6.048).

By contrast, early‐stage melanoma patients who develop metastasis during the follow‐up had lower DCD values at the moment of diagnosis than the group of patients that remained disease‐free (Fig. 2 *c*). A REPTree was constructed using a tenfold cross‐validation test for a population of *N* = 271 instances (the total number of stage I and II instances). For the case of melanoma stage II, DCD was the most effective descriptor to obtain a proper classification between metastatic or nonmetastatic responses, with 80.07% correctly classified instances. In the tree (Fig. 2 *d*), first numbers in parentheses are the number of instances satisfying each rule and their sum is the total number of instances (184 + 46 + 41 = 271). Second numbers in parenthesis are the number of instances that were incorrectly classified for each rule, and their sum (46 + 5 + 3 = 54) is equal to the total number of incorrect instances. Thus, the sum of the second numbers divided by the sum of the first ones determines the algorithm accuracy (*i.e*., 1 – 54/271 = 0.8007). Accordingly, the REPTree algorithm revealed that patients with a stage II melanoma and serum levels of DCD <2.9 μg/ml will develop metastasis during follow‐up, with an accuracy of 80.07% correctly classified instances.

Finally, to validate which factors might be predictive of metastatic progression in melanoma, we used a logistic regression model. Only patients with AJCC stages I and II of the disease met the requirements for analysis with such a method, and the factors considered were age, sex, stage, disease‐free interval and the serum levels of SAA, CLU, PG, VN and DCD. A logistic regression model was used to determine the most informative combination of independent factors for prognosis and it showed that stage (*p* = 0.000), and serum VN (*p* = 0.037) or DCD (*p* = 0.025) levels were significantly associated with the metastatic progression of melanoma (Table 4). Therefore, stage, DCD and VN serum levels are risk factors for the malignant progression of melanoma in patients diagnosed at early stages of the disease.

## Discussion

Several specific or nonspecific serological biomarkers can be found in advanced melanoma patients.^9^ LDH as well as S100B serum levels have been correlated with poor prognosis in AJCC stage III–IV melanoma patients^3^; however, the poor sensitivity and specificity of those markers limit their use in early melanoma patients (AJCC stage I–II). Thus, there are still no prognostic factors that are suitable to use at early stages of tumor development.^2^, ^15^ Accordingly, the aim of this study was to discover serological markers that can be used at the time of primary melanoma diagnosis to identify those early‐stage melanoma patients with a high risk of developing metastasis during follow‐up.

Although patients diagnosed at the early stages of the disease have a better prognosis and a high survival rate,^1^ there are a percentage of them who develop metastases during the first 2 years of follow‐up after surgical removal of the primary tumor. In our study, 25% of the early‐stage melanoma patients developed metastasis during the first 2 years of follow‐up and a high proportion of them (65.7%) developed distant metastases, reflecting the aggressiveness and invasiveness of the tumor. To discover new serum proteins associated with the malignant progression of melanoma, serum samples from ten stage IIA patients were analyzed using a proteomic approach. Of these, five samples were from stage IIA patients that remained disease‐free during a 10‐year follow‐up and they were compared with sera from five stage IIA patients who developed distant metastasis during the first 2 years of follow‐up. A total of 42 proteins were seen to be different in these samples, almost half of which were involved in immune and inflammatory responses (43%), while the next most represented category were proteins with metabolic and apoptotic functions (13%). These cellular processes are each closely associated with tumor progression.

Five proteins related to immune and inflammatory responses (SAA, CLU), cell adhesion (PG and VN) and antimicrobial activity (DCD) were selected for validation as prognostic serum biomarkers in a group of 348 melanoma patients using ELISA. The results indicated that the serum levels of these proteins were significantly different between healthy controls and melanoma patients; however, only VN and DCD seem to be associated with the metastatic progression of these tumors.

Significantly higher serum levels of SAA and CLU apolipoproteins were detected in melanoma patients than in healthy controls, although no relation with tumor stage and metastatic progression was evident. SAA protein is mainly produced by the liver and it is associated with acute‐phase inflammatory responses.^16^ Elevated SAA serum levels have been observed in different tumors, including melanoma,^17^, ^18^, ^19^ which may be due to the inflammatory reactions and tumor necrosis associated with malignant disease. Moreover, SAA protein has also been seen to be expressed by some tumor cells, suggesting that it is directly involved in tumor progression.^20^ In contrast to our results, SAA has been proposed to be a serum prognostic factor for early‐stage melanoma patients,^17^ a discrepancy that could be due to a different model design (a different set of variables were considered to perform the analysis) together with a different classification of tumor stages. While in this earlier study AJCC stage I, II and III were considered as early stages of melanoma, we considered AJCC stage I and II as early stages of the disease. Indeed, by stage III there is already lymphatic spread and hence, we considered AJCC stage III and IV as advanced stages. Interestingly, recent studies showed that SAA can stimulate immunosuppression in melanoma patients, inducing the production of interleukin‐10 (IL‐10) to suppress cell immunity.^21^ Accordingly, elevated serum levels of IL‐10 have been related to a worse prognosis of melanoma.^22^, ^23^

The CLU protein is thought to be involved in processes such as carcinogenesis, tumor progression, immune system regulation and apoptosis,^24^ and it is expressed strongly in different tumor types.^25^, ^26^, ^27^ Moreover, in colon carcinoma^28^ and prostate cancer,^29^ the CLU levels reflect disease progression and the biological aggressiveness of the tumor. Although serum CLU levels increased with tumor stage in our group of melanoma patients and it seems to be associated with advanced tumor stages, the multivariate analysis did not show any clear relationship with metastatic progression in early stage I–II melanoma patients (Table 4).

**Table 4:** Multivariate analysis of the prognostic factors in AJCC stage I–II patients with melanoma

Covariates	Logistic regression (*p* values)
Age	0.469
Sex	0.272
Stage	0.000^a^
[SAA]	0.177
[CLU]	0.290
[PG]	0.916
[VN]	0.037^a^
[DCD]	0.025^a^

a Significant differences, *p* < 0.05.

Adhesion molecules are strong candidates to be involved in the metastatic spread of melanoma.^30^ PG, also known as γ‐catenin,^31^, ^32^ is a component of adherens junctions and desmosomes, and it plays an important role in Wnt/β‐catenin signaling.^33^, ^34^ In several tumors, the loss of PG expression by cells has been related to tumor growth, invasive behavior and hence metastatic progression.^35^, ^36^ However, while we found that serum PG levels are significantly higher in melanoma patients than in healthy controls, they were not significant different with regard to tumor stage and metastatic progression. By contrast, VN serum concentration is associated to a poor outcome in patients and metastasis progression. This adhesion protein has also been identified as a serum marker in other types of tumors including breast cancer^37^ and hepatocellular carcinoma.^38^ VN is a glycoprotein involved in cell adhesion, extracellular matrix remodeling and in tumor cell migration, principally through its interaction with integrins, the urokinase‐type plasminogen activator receptor (uPAR) and plasminogen activator inhibitor‐1 (PAI‐1).^39^ VN has not been studied as a serum marker associated with melanoma prognosis to date; however, VN receptor expression on melanoma cells has been associated with poor disease prognosis.^15^, ^40^ Indeed, serum VN might regulate the differentiation of cancer stem cells and promote tumorigenesis through its integrin αVβ3 receptor,^41^ an interaction that upregulates MMP‐2 expression and leads to degradation of the stroma.^42^

Our results show that early‐stage melanoma patients with high levels of VN were 2.8 times more likely to develop metastases during follow‐up. By stage II, when the tumor is no longer limited to the epidermis, higher serum VN levels might be due to the invasion of the dermis that involves the destruction of the basement membrane and extracellular matrix, potentially releasing different components like VN into the serum. Recently, VN was considered to play an important role in the regulation of endothelial permeability, facilitating tumor migration, while its binding to its receptor enhances VEGF signaling, thereby promoting angiogenesis and vascular permeability.^43^

DCD serum levels are elevated in melanoma patients with respect to the healthy controls, although in the early‐stage patients who develop metastasis during follow‐up there is a significant decrease in DCD levels. Indeed, applying our classification methods to AJCC stage I–II patients revealed that the stage II patients with DCD levels <2.98 μg/ml are more likely to develop metastasis during follow‐up (80% correctly classified instances). Elevated DCD levels have also been found in the serum of breast cancer patients collected at the time of diagnosis,^44^ although DCD expression in these tumor cells is associated with advanced clinical stage and poor disease prognosis.^45^ By contrast, in our group of melanoma patients, lower level of DCD was a significant factor that predicted a metastatic outcome in AJCC stage II patients. Hence, DCD could be a potent biomarker of prognosis that might improve personalized medical care and the survival of early‐stage melanoma patients.

Although there is evidence that DCD participates in the host's defense,^46^ its role in cell survival and carcinogenesis and the functional importance of the peptides derived from this protein are not clear.^45^ Posttranslational modifications of DCD produce peptides with different functions: survival‐promoting peptide and the antimicrobial DCD‐1 protein. In addition, tumor cells can generate another peptide called proteolysis‐inducing factor (PIF) that is associated with cachexia in cancer patients.^47^ Although it is known that melanoma cells express DCD,^48^, ^49^ until now this protein has not been associated with malignancy. The multivariate analysis of AJCC I–II melanoma patients carried out here demonstrates the significant association between serum DCD levels and poor disease prognosis. Indeed, our results show that patients in stage II melanoma with DCD levels lower that 2.98 will likely have a metastatic progression.

Finally, in patients with melanoma *in situ* that never develop metastasis, serum CLU, VN and DCD levels were similar to those in the controls and only the serum SSA and PG levels differed. However, in AJCC III–IV stage patients who have a worse survival, there were slightly higher levels of the proteins analyzed. These differences may be due to the small number of stage III–IV patients studied or because body homeostasis in patients with metastatic spread would differ from that in patients with a local skin lesion. To classify these issues, a further analysis based on a larger prospective study will be initiated in the near future.
